# Functional Connectome Predicts Cognition and Links White Matter Hyperintensity Burden to Cognitive Impairment Across the Vascular Cognitive Impairment Continuum

**DOI:** 10.3390/brainsci16070695

**Published:** 2026-06-30

**Authors:** Haoying He, Yifan Fang, Jiu Jiang, Dongwei Lu, Linna Ji, Bihan Liu, Yuxiang Jiang, Jing Cao, Bin Mei, Junjian Zhang

**Affiliations:** 1 Department of Neurology, Zhongnan Hospital of Wuhan University, 169# East Lake Road, Wuchang District, Wuhan 430071, China; hehaoying@whu.edu.cn (H.H.); 2014302180045@whu.edu.cn (Y.F.); linnaji@whu.edu.cn (L.J.); liubihan@whu.edu.cn (B.L.); 2025183039036@whu.edu.cn (Y.J.); neuromei20@whu.edu.cn (B.M.); 2 Hubei Provincial Clinical Research Center for Dementia and Cognitive Impairment, 169# East Lake Road, Wuhan 430071, China; 2008302180101@whu.edu.cn (D.L.); zn002468@whu.edu.cn (J.C.); 3 Electronic Information School, Wuhan University, 299# Bayi Road, Wuchang District, Wuhan 430064, China; jiangjiu@whu.edu.cn; 4 Department of Neuropsychology, Zhongnan Hospital of Wuhan University, 169# East Lake Road, Wuchang District, Wuhan 430071, China

**Keywords:** vascular cognitive impairment, white matter hyperintensity, resting-state functional MRI, connectome-based predictive modeling, network strength

## Abstract

**Highlights:**

**What are the main findings?**

- Whole-brain connectome-based predictive modeling identified functional connectivity patterns related to global cognition and TMT-B performance across the VCI continuum.
- The TMT-B-related network was enriched in weak between-network connections, and its cross-validated network strength statistically linked WMH burden to cognitive impairment.

**What are the implications of the main findings?**

- Functional connectome patterns may capture clinically meaningful variability in cognition across the vascular cognitive impairment continuum.
- Distributed functional disconnection statistically links WMH burden to cognitive impairment in VCI.

**Abstract:**

**Background**: White matter hyperintensity (WMH) is a hallmark of cerebral small vessel disease and an important contributor to vascular cognitive impairment (VCI), yet lesion burden incompletely explains interindividual variability in cognitive outcomes across the VCI continuum. Functional connectome signatures relevant to this variability remain incompletely characterized. **Methods**: We analyzed multicenter resting-state functional MRI data from 247 participants spanning vascular risk factors with normal cognition, vascular mild cognitive impairment, and vascular dementia. Exploratory external testing was performed in an independent dataset of 37 participants. Connectome-based predictive modeling (CPM) with permutation testing was used. To reduce circularity, we computed cross-validated network strength (cvNS) using predictive masks defined within training folds only. **Results**: We identified cross-validated functional connectivity patterns associated with MoCA (positive model, *p_perm* = 0.034) and TMT-B performance (negative model, *p_perm* = 0.001). These patterns were most prominently represented in Frontoparietal, Motor, and Subcortical–Cerebellar regions. The TMT-B negative network showed a substantial contribution from weak between-network connections (64.4% of predictive edges). In the external dataset, network strength computed from discovery consensus masks remained associated with MoCA. Greater WMH volume was associated with worse TMT-B performance, and mediation analyses indicated that cvNS computed from the TMT-B connectivity pattern statistically linked WMH volume to both TMT-B (indirect = 0.090, 95% CI [0.018, 0.225], *p* = 0.023) and MoCA performance (indirect = −0.007, 95% CI [−0.019, −0.001], *p* = 0.031). **Conclusions**: CPM-derived functional connectivity patterns capture meaningful continuous variability in cognition across the VCI continuum and provide statistical support consistent with WMH-related functional disconnection as a network-level correlate of cognitive impairment.

## 1. Introduction

Vascular cognitive impairment (VCI) encompasses a spectrum of cognitive deficits, ranging from mild cognitive impairment (VaMCI) to vascular dementia (VaD), driven by cerebrovascular pathologies and the cumulative burden of vascular risk factors (VRFs) [1,2,3]. As the second most common cause of dementia worldwide, VCI represents a major public health challenge, particularly given the aging population and the high prevalence of VRFs [4]. VCI is pathologically heterogeneous and unfolds along a clinical continuum, often beginning in cognitively normal individuals with VRFs (VRF-CN).

Current diagnosis and monitoring in VCI are largely based on structural neuroimaging [2,3,5,6]. White matter hyperintensity (WMH), a hallmark of cerebral small vessel disease, reflects white matter demyelination and axonal loss [5,6]. Advanced structural techniques, such as diffusion tensor imaging (DTI), are sensitive to white matter microstructural abnormalities in VCI [5,6,7,8,9]. The prevailing disconnection hypothesis posits that such structural damage disrupts the brain’s communication highways, particularly Prefrontal–Subcortical circuits [9,10,11]. Neuroimaging evidence increasingly supports the view that VCI is a brain network disorder, shifting the focus from local lesions to a more global, network-level perspective [1,12]. Consistent with this framework, structural connectome studies have demonstrated that the disruption of network topology, particularly involving hub regions, is associated with cognitive impairment and may partially explain interindividual variability in cognitive outcomes [13,14,15,16]. However, structural markers are also common in normal aging and other neurodegenerative diseases, and lesion burden does not always show a linear relationship with cognitive severity, likely because strategically located lesions may have disproportionate effects [1,2,3,5,17,18,19]. Together, these observations suggest that structural measures capture only part of the disease process and that the brain’s functional response, specifically its ability to maintain or lose network integration, plays a decisive role in the clinical outcome [9,20].

Functional MRI (fMRI) provides a complementary view into the functional consequences of vascular pathology. Previous fMRI studies have demonstrated that VCI patients exhibit aberrant functional connectivity (FC), particularly within the Default Mode Network (DMN) [21,22]. Nevertheless, the literature relying on group-level statistical comparisons or a priori regions of interest has several limitations. On the one hand, group-level approaches cannot adequately capture differences in individual disease severity and pathological burden, particularly the heterogeneity that exists within the same clinical diagnosis [23,24]. On the other hand, even focal vascular pathology may lead to widespread alterations in whole-brain FC [20]. Evidence suggests that distributed whole-brain functional activity patterns may better capture interindividual cognitive heterogeneity than analyses restricted to localized regions of interest [25]. Moreover, the functional connectome exhibits stable and individually specific patterns [24], and may be more sensitive to connectome pathology than tractography-derived structural connectivity measures [23]. These features make the whole-brain functional connectome particularly attractive for characterizing individual differences across the VCI continuum. However, their use for studying individual variability across the VCI spectrum remains limited.

In this study, we applied connectome-based predictive modeling (CPM) to resting-state fMRI (rs-fMRI) data from a multicenter VCI dataset [24,26]. CPM is a whole-brain FC framework that uses cross-validation to identify predictive connectivity patterns from participant-level FC matrices, and has been used to model individual differences in fluid intelligence, neuropsychiatric conditions, and other behavioral features [27]. Our aim was to identify topologically interpretable FC signatures associated with cognition across the VCI continuum and to determine whether these patterns could predict cognitive performance at the individual level. We further tested whether network strength computed from the identified FC patterns statistically links WMH burden to domain-specific cognition within a mediation framework.

## 2. Materials and Methods

### 2.1. Participants of the Discovery Dataset

This multicenter study utilized baseline multimodal data from the Wuhan Zhongnan VCI cohort, in which a total of 487 participants were enrolled. Participants were consecutively recruited between November 2019 and June 2024 from the inpatient and outpatient clinics of the Department of Neurology and the Department of Neuropsychology at Zhongnan Hospital of Wuhan University (Wuhan, Hubei Province), and from the Shuiguohu Community in Wuchang District. For each participant, multimodal data were collected, including demographics, neuropsychological assessments, laboratory test results, and multimodal MRI.

The inclusion criteria were as follows: (1) age between 50 and 80 years; (2) at least primary education (equivalent to 6 years); (3) ability to cooperate in completing the neuropsychological assessments and MRI examinations; (4) patients with VCI that met the diagnostic criteria of the Vascular Impairment of Cognition Classification Consensus Study (VICCCS) [28] and the Chinese Guidelines for the Diagnosis and Treatment of Vascular Cognitive Impairment (2019) [29], defined as an objectively verified cognitive impairment in the presence of cerebrovascular disease (including ischemic/hemorrhagic injury, large/small vessel disease, or hypoperfusion) or associated vascular risk factors; (5) cognitively normal (CN) participants who reported no cognitive complaints, showed no objective impairment on neuropsychological assessments, and had vascular and cognitive risk factors [30,31]; (6) patients who signed the informed consent. The diagnosis of VCI was determined by two experienced senior neurologists.

**Figure 1:**
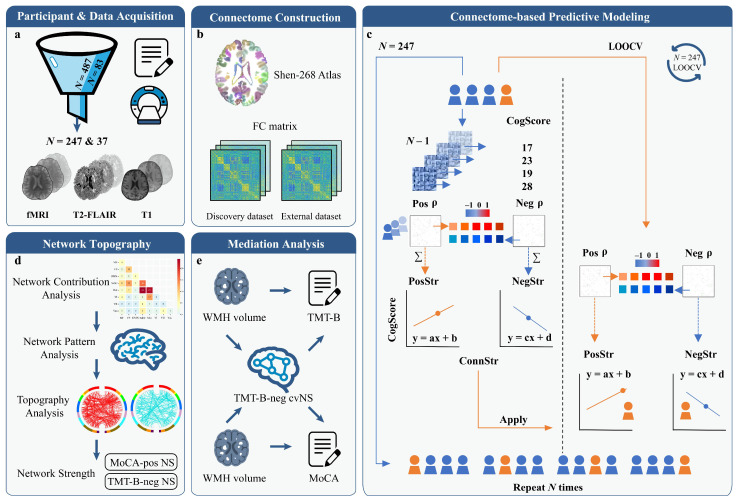
Study design and analytical framework. (**a**) Participant selection and multimodal data acquisition. Recruitment of the discovery (*N* = 247) and external (*N* = 37) datasets, involving multimodal MRI (fMRI, T1, T2-FLAIR) and neuropsychological assessments. (**b**) Functional connectome construction. Whole-brain functional connectivity matrices were derived using the Shen-268 atlas for both datasets. (**c**) Connectome-based predictive modeling (CPM) framework. Schematic illustration of the CPM procedure, showing the training phase (**left**) and testing phase (**right**) under leave-one-out cross-validation. (**d**) Network topography and network strength derivation. Predictive connectivity patterns identified by CPM were visualized topographically, and cross-validated network strength measures were derived for MoCA-pos and TMT-B-neg model. (**e**) Mediation analysis. Mediation models were used to examine whether TMT-B-neg cvNS statistically mediated the association between white matter hyperintensity volume and cognitive performance. Abbreviations: CPM, connectome-based predictive modeling; LOOCV, leave-one-out cross-validation; cvNS, cross-validated network strength; TMT-B-neg cvNS, cross-validated network strength of the TMT-B-neg predictive network; TIV, total intracranial volume; WMH-v, white matter hyperintensity volume.

The exclusion criteria were: (1) a history of stroke within the past three months; (2) other major central neurological diseases; (3) other systemic diseases that may contribute to cognitive impairment; (4) severe visual or hearing impairments that would interfere with assessments; (5) MRI scans with significant motion artifacts. The overall study design and analytical framework are illustrated in Figure 1.

### 2.2. Neuropsychological and Functional Assessments

Comprehensive neuropsychological functional assessments were conducted by experienced neuropsychologists, all of whom had received standardized training prior to the study’s initiation. The battery included the Montreal Cognitive Assessment (MoCA) [32], the Mini-Mental State Examination (MMSE) [33], Trail-Making Test A (TMT-A), Trail-Making Test B (TMT-B) [34], the Activities of Daily Living (ADL) scale [35], the Hamilton Anxiety Rating/Hamilton Depression Rating Scale (HAMA/HAMD) [36,37], the Boston Naming Test (BNT-15) [38] or Verbal Fluency Test (VFT-3 min), and the Chinese Auditory Verbal Learning Test (CAVLT) [39].

### 2.3. Independent External Dataset

To assess the connectivity patterns and network strength derived from CPM in an exploratory external setting, we included an independent external dataset of 83 participants. These participants were consecutively recruited from the Department of Neurology at Xiangyang Central Hospital between January 2023 and July 2024. This dataset included patients with acute mild ischemic stroke, with MRI scans obtained within 48 h and cognitive assessments completed within 3–5 days. Therefore, this analysis was considered an exploratory external test rather than strict external validation.

### 2.4. MRI Data Acquisition

Multimodal MRI data, including T1-weighted, T2-Fluid-Attenuated Inversion Recovery (T2-FLAIR), and rs-fMRI, were acquired using 3 T scanners across centers. The discovery dataset was scanned at Zhongnan Hospital of Wuhan University using a SIEMENS Trio Tim (Siemens Healthineers, Erlangen, Germany) and a GE 750 W system (GE Healthcare, Milwaukee, WI, USA). The external set was scanned at Xiangyang Central Hospital using a GE SIGNA Architect system (GE Healthcare, Milwaukee, WI, USA). Detailed acquisition parameters for all protocols are provided in the Supplementary Materials.

### 2.5. Imaging Preprocessing and Functional Connectivity Network Construction

rs-fMRI data were preprocessed using SPM12 [40] and DPABI [41] V8.2. All structural and functional images were visually inspected for artifacts and data quality and were manually reoriented to the anterior commissure–posterior commissure (AC-PC) line.

The processing pipeline included removing the first five time points, slice timing correction, head motion correction, linear detrending, and nuisance covariate regression. Regressors included the Friston 24-parameter motion model, white matter and cerebrospinal fluid signals, and the global signal, which was included to reduce widespread physiological and motion artifacts [42]. Specifically, motion artifacts were mitigated by regressing out volumes with framewise displacement (FD) > 0.5 mm, together with one preceding and two subsequent volumes. High-resolution T1-weighted images were co-registered to the mean functional image generated after motion correction. These T1 images were then segmented and normalized to Montreal Neurological Institute (MNI) space using unified segmentation algorithm and Diffeomorphic Anatomical Registration Through Exponentiated Lie algebra (DARTEL). The resulting transformation parameters were then applied to the motion-corrected functional volumes. Images were spatially smoothed using a 6 mm full-width at half-maximum (FWHM) Gaussian kernel and temporally bandpass filtered (0.01–0.1 Hz). To construct the functional connectome, the brain was parcellated into 268 nodes using the Shen-268 functional atlas [43], as a resolution of 200 to 300 regions has been recommended for robust modeling [26]. FC was calculated as the Pearson correlation between nodal time series, resulting in a 268 × 268 FC matrix for each participant.

The fMRI data for this cohort were acquired on two MRI systems, one of which underwent a scanning protocol upgrade during the study period, resulting in three scanning protocols. Such multi-scanner and multi-protocol data are subject to non-biological variance related to scanners and acquisition differences [44]. Although we attempted to standardize our acquisition protocols and apply similar parameters, this procedure alone cannot fully eliminate inter-scanner differences [45]. To reduce these effects, we used a novel rs-fMRI harmonization method based on a Riemannian framework [46]. FC matrices were harmonized within each leave-one-out cross-validation (LOOCV) iteration using the Bures–Wasserstein Riemannian framework with ComBat correction and the identity matrix as the reference barycenter. The harmonization parameters were estimated exclusively from the training participants, and the resulting transformation was applied to the held-out participant. These harmonized matrices were then Fisher-z transformed and used for the subsequent CPM analyses. To validate the effectiveness of harmonization, we performed a site-effect analysis before and after harmonization. Specifically, we used a covariate-adjusted PERMANOVA to test whether site explained inter-subject variation in connectome features after accounting for age, sex, and education. Statistical significance was assessed using a Freedman–Lane permutation procedure. For the post-harmonization analysis, out-of-fold harmonized features from the LOOCV procedure were used to avoid information leakage.

### 2.6. Connectome-Based Predictive Modeling 

We applied CPM (Figure 1c) to predict cognitive function from the fold-wise harmonized FC matrices using an LOOCV framework across the VCI continuum [26]. In each LOOCV iteration, we correlated every edge in the 268 × 268 harmonized training matrices with the behavioral variable using Spearman correlation (chosen due to non-normal distribution; Kolmogorov–Smirnov test, *p* < 0.05). Edges were selected at a significance threshold of *p* < 0.01 and separated into positive and negative networks. Network strength was calculated for each participant by summing the strength values of the selected edges. These summary statistics were entered into linear regression models to predict the behavioral score of the held-out subject. This entire process was iterated until every participant had served as the test subject. In the CPM construction, age, sex, and education were included as covariates. Model significance was assessed with a 5000-permutation test by shuffling behavioral variables. Predictive performance was evaluated using Spearman correlation (*ρ*) and the coefficient of determination (R^2^) between predicted and observed values. Five cognitive measures were evaluated, with positive and negative CPM models assessed separately. Each model addressed a separate model-specific association and was not treated as a surrogate test of a single omnibus null hypothesis concerning cognition in general. Therefore, no across-model multiplicity adjustment was applied [47,48]. The reported permutation *p* values are model-specific and unadjusted across cognitive outcomes and CPM models. All model results are reported and interpreted as nominal, hypothesis-generating evidence. All analyses were performed using MATLAB 2022a.

### 2.7. Head Motion Control

Head motion was controlled at both preprocessing and modeling stages. During preprocessing, volumes with FD-Power > 0.5 mm, together with one preceding and two subsequent volumes, were modeled as nuisance regressors. At the participant level, participants were excluded if maximum translational displacement > 2 mm, rotational motion > 2°, or mean FD-Jenkinson > 0.2 mm. Data quality was further assessed by calculating the percentage of volumes with FD-Power > 0.5 mm for each participant.

To further assess the robustness of our findings to head motion, we conducted sensitivity analyses during the modeling stage. Specifically, we first confirmed that the behavioral variables were not significantly correlated with mean FD-Jenkinson. We then rebuilt the CPMs with mean FD-Jenkinson included as a covariate. Only models that remained significant and showed high edge overlap with the corresponding original models were kept for subsequent analyses. Edge overlap was quantified as the proportion of significant FC edges from the original model that were also identified in the motion-corrected model.

### 2.8. Network Topography Analysis

To define the final connectivity pattern for topological analysis, we retained only those edges that were consistently selected in every LOOCV iteration. These edges were designated as consensus edges, and the resulting overall pattern was designated as the consensus network. All subsequent topological analyses were based on this consensus edge set.

To characterize the topology of the consensus network, the 268 nodes of the Shen atlas were mapped to 10 anatomical lobes [43] and 8 canonical functional networks [24]. Anatomically, the nodes were assigned to 10 brain regions/lobes: Prefrontal cortex (46 nodes), Motor cortex (21 nodes), Insula (7 nodes), Parietal cortex (27 nodes), Temporal cortex (39 nodes), Occipital cortex (25 nodes), Limbic lobe (36 nodes), Cerebellum (41 nodes), Subcortical areas (17 nodes), and Brainstem (9 nodes). Functionally, the nodes were assigned to eight networks: Medial Frontal (MF, 29 nodes), Frontoparietal (FP, 34 nodes), Default Mode Network (DMN, 20 nodes), Subcortical Cerebellum (SubC, 90 nodes), Motor (Mot, 50 nodes), Visual I (VI, 18 nodes), Visual II (VII, 9 nodes), and Visual Association (Vas, 18 nodes).

Based on the consensus network, we further examined its topological organization. Nodal degree centrality was calculated for each node on the binarized consensus network as the number of consensus edges connected to that node, and nodes with the highest degree were defined as network hubs. The contribution of each functional network was quantified using both the absolute number of consensus edges and a normalized connection ratio, which controls for network size and was defined as the number of observed edges divided by the total number of possible edges for the corresponding functional network or network pair. Finally, consensus edges were classified as within-network or between-network connections for pairwise network analysis across the eight canonical functional networks.

### 2.9. Network Virtual Lesion and Inclusion Analysis

To quantify the contribution of specific canonical functional networks, we performed virtual lesion and inclusion analyses [24,49,50]. In the lesion analysis, nodes belonging to a target functional network were systematically removed from the connectivity matrix before rerunning the CPM pipeline. For example, lesioning the Mot (50 nodes) reduced the 268 × 268 matrix to a 218 × 218 matrix. Conversely, inclusion analysis involved building models exclusively using nodes from a single target functional network [24]. This procedure was repeated for each of the eight canonical functional networks. The standard CPM pipeline was then reapplied to these modified matrices, and predictive performance was compared with that of the corresponding whole-brain model.

### 2.10. Definition and Calculation of Network Strength Derived from CPM

To characterize the predictive edges, we analyzed the distribution of FC strengths across predictive edges using Kernel Density Estimate (KDE) plots, separately for within- and between-network connections. For the predictive masks identified by CPM, network strength (NS) was defined as the sum of each participant’s FC values across all edges included in that mask. This measure was used as a participant-level summary of connectivity within the predictive mask derived from CPM. Positive and negative predictive masks were analyzed separately. To reduce circularity within dataset inference, all brain behavior analyses were based on cross-validated network strength (cvNS). Specifically, in each LOOCV fold, predictive edges were selected using training data only, and the resulting masks were then applied to the held-out participant to compute out-of-fold network strength. Finally, in an exploratory external analysis, we derived a consensus mask of edges selected in 100% of LOOCV folds in the discovery dataset and applied this fixed mask unchanged to compute network strength for each participant in the independent external dataset.

### 2.11. Statistical Analysis of Network Strength Derived from CPM

All inferential analyses in the discovery dataset were performed using the cvNS to reduce circularity. Associations between cvNS and cognitive performance were assessed using partial Spearman correlations controlling for age, sex, and education. Data normality was assessed using the Shapiro–Wilk test and visual inspection.

Group comparisons were conducted in two steps. First, differences in cvNS between the VRF-CN and VCI groups were analyzed using independent *t*-tests or Mann–Whitney U tests, depending on data distribution. Second, differences across the three subgroups (VRF-CN, VaMCI, and VaD) were assessed using the Kruskal–Wallis test.

Subsequently, WMH volume was calculated using WMH-seg, our previously developed automatic segmentation tool [51]. To reduce scanner variance, WMH volumes were harmonized using neuroHarmonize [52], with age, sex, education, and total intracranial volume (TIV) included as covariates [53]. TIV was estimated using the CAT12 toolbox [54]. All subsequent WMH-related analyses were performed using the harmonized WMH values and were adjusted for age, sex, education, and TIV. Mediation analysis with 5000 bootstrap iterations was performed to test whether cvNS mediates the relationship between WMH volume and cognition.

For exploratory external analysis, the consensus masks derived from the discovery dataset were applied to the external dataset to compute NS values. Partial Spearman correlations were performed between NS and cognition controlling for age, sex, and education. WMH-related analyses were not performed in the external dataset due to limited T2-FLAIR availability. Statistical analyses were conducted using SPSS 20, SciPy v1.10, and Pingouin v0.5.5.

## 3. Results

### 3.1. Participants

**Figure 2:**
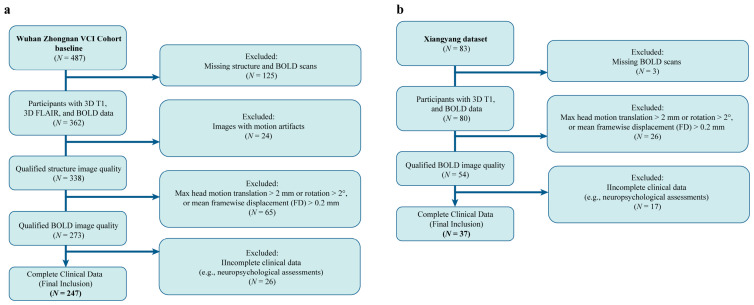
Flowchart of participant enrollment. (**a**) Discovery dataset (Wuhan Zhongnan VCI cohort baseline): Illustration of the selection process. Initially, 487 participants were screened, and 247 were included in the final analysis after applying clinical and imaging exclusion criteria. (**b**) Independent external dataset (Xiangyang dataset): Selection process for the independent dataset. Initially, 83 participants were screened, and 37 were included in the final analysis. Abbreviations: VCI, vascular cognitive impairment; BOLD, Blood Oxygen Level-Dependent; FD, framewise displacement.

Initially, 487 participants were screened; ultimately, 247 met all inclusion criteria and were included in the subsequent analyses. A detailed flowchart of participant enrollment is shown in Figure 2a. The demographic and clinical characteristics of the total dataset, as well as the VCI and VRF-CN subgroups, are summarized in Table 1.

For the external dataset, 83 participants were screened; ultimately, 37 met all criteria and were retained for analysis. A detailed enrollment flowchart is shown in Figure 2b, and demographics are summarized in Supplementary Table S1.

### 3.2. Predictive Performance Using CPM

Before harmonization, site significantly explained connectome variation after adjustment for age, sex, and education; F = 2.81, partial R^2^ = 0.0228, and *p* < 0.001. After harmonization, the site effect was substantially reduced and no longer significant; F = 0.41, partial R^2^ = 0.003, and *p* = 1.000.

We then applied CPM to predict cognitive performance from whole-brain FC, controlling for age, sex, and education. As all behavioral variables were non-normally distributed (Kolmogorov–Smirnov test, *p* < 0.05; Supplementary Table S2), we employed Spearman correlation.

Initially, three models showed statistical significance: (1) MoCA-pos: predicted by a positive network of 223 consensus edges (ρ = 0.202, R^2^ = 4.64%, *p_perm* = 0.034); (2) MoCA-neg: predicted by a negative network of 205 consensus edges (ρ = 0.193, R^2^ = 3.48%, *p_perm* = 0.048); (3) TMT-B-neg: predicted by a negative network of 163 consensus edges (ρ = 0.260, R^2^ = 6.03%, *p_perm* = 0.001). Models for other cognitive assessments (MMSE, TMT-A, and CAVLT) were not significant (Supplementary Table S3). These initial results indicate that whole-brain FC can significantly predict global cognition (MoCA) and executive-attentional function (TMT-B) in the VCI continuum.

We performed several quality control analyses to ensure our results were not confounded by head motion. First, mean FD-Jenkinson showed no significant correlation with behavioral scores (*r* < 0.1, *p* > 0.05; Supplementary Table S4). Additionally, across the 247 participants, the number of volumes with FD-Power > 0.5 mm ranged from 0 to 78 (mean = 6.05, median = 2). As a percentage of total volumes, this ranged from 0% to 33.19% (mean = 2.57%, median = 0.85%). Next, we re-ran the CPMs adding mean FD as a covariate. Under this stricter control, the MoCA-neg model lost significance (*p_perm* = 0.061) and was excluded from further analysis. Conversely, both the MoCA-pos (*p_perm* = 0.040) and TMT-B-neg (*p_perm* = 0.034) models remained significant. Crucially, the network patterns identified in the motion-controlled models showed high edge overlap (Supplementary Table S5) with the original models: 97.31% for MoCA-pos and 88.96% for the TMT-B-neg model. This high stability confirms that these predictive networks are not driven by head motion artifacts. Consequently, we proceeded with the two models: MoCA-pos and TMT-B-neg.

### 3.3. Network Connectivity Patterns

**Figure 3:**
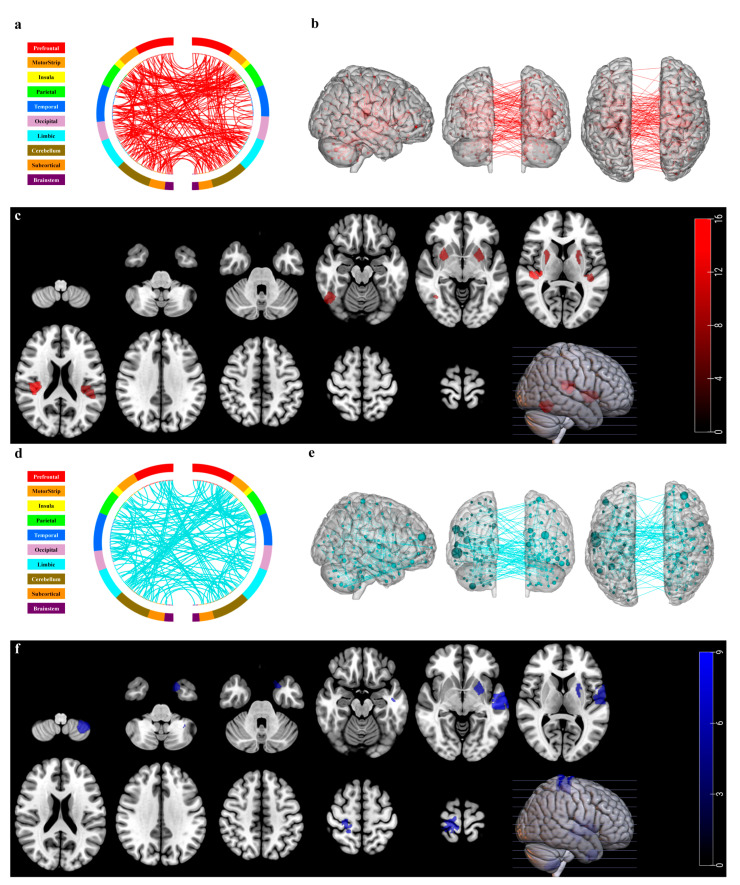
Topographical visualization of predictive connectivity patterns. (**a**–**c**) MoCA-pos network, comprising 223 consensus edges (red lines). (**a**) Chord diagram showing the topological distribution of the network. The outer ring is color-coded according to ten macroscale brain regions in each hemisphere, and the inner lines indicate the consensus edges. (**b**) 3D brain surface rendering of the spatial distribution of the network. (**c**) MNI152 slice views showing the anatomical locations of the five nodes with the highest degree centrality; the color bar denotes node degree. (**d**–**f**) TMT-B-neg network, comprising 163 consensus edges (blue lines). (**d**) Chord diagram showing the topological distribution of the network. (**e**) 3D brain surface rendering of the spatial distribution of the network. (**f**) MNI152 slice views showing the anatomical locations of the five nodes with the highest degree centrality. Abbreviations: MoCA, Montreal Cognitive Assessment; TMT-B, Trail-Making Test Part B; MNI152, Montreal Neurological Institute 152 template.

Regarding anatomical distribution, the MoCA-pos network (Figure 3a–c) exhibited a dense, widespread inter-hemispheric architecture. High-degree hubs were identified in the right Primary Auditory Cortex (BA 41), right Fusiform Gyrus (BA 37), Putamen, and left Supramarginal Gyrus (BA 40). Similarly, the TMT-B-neg network (Figure 3d–f) was anchored by hubs in the left Superior Temporal Gyrus (BA 22), right Postcentral Gyrus (BA 1), left Premotor Cortex (BA 6), Putamen, and Cerebellum.

**Figure 4:**
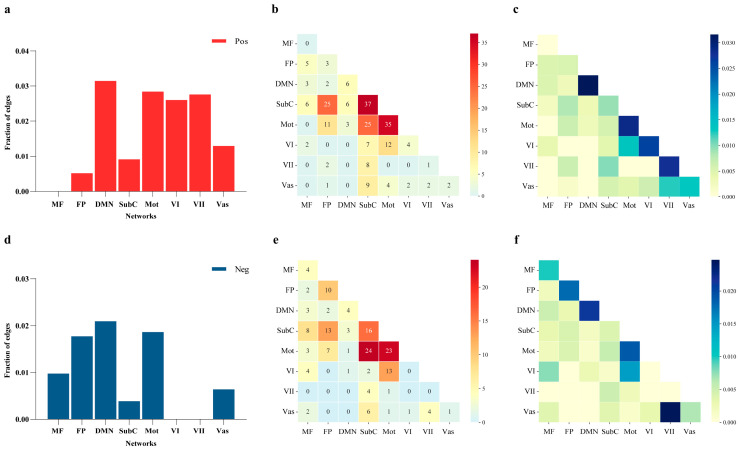
Functional network topography of predictive connectivity patterns. Consensus edges identified from each predictive model were summarized across eight canonical functional networks. (**a**–**c**) MoCA-pos network. (**a**) Normalized ratios of within-network connections for each functional network. (**b**) Pairwise heatmap matrix of absolute number of consensus connections. Values indicate the number of edges. (**c**) Pairwise heatmap matrix of normalized connection ratios adjusted for the number of nodes in each network (network size). (**d**–**f**) TMT-B-neg network. (**d**) Normalized ratios of within-network connection for each functional network. (**e**) Pairwise matrix of absolute connection counts. (**f**) Pairwise matrix of normalized connection ratios adjusted for network size. Abbreviations: MoCA, Montreal Cognitive Assessment; TMT-B, Trail-Making Test Part B; MF, Medial Frontal; FP, Frontoparietal; DMN, Default Mode Network; SubC, Subcortical Cerebellum; Mot, Motor; VI, Visual I; VII, Visual II; Vas, Visual Association.

From a functional perspective, absolute connection counts for the MoCA-pos network (Figure 4a–c) were concentrated in SubC and Mot networks. However, after controlling for network size, the DMN showed the highest normalized within-network connectivity, followed by Mot and visual (VI/VII) networks. Conversely, the TMT-B-neg network (Figure 4d–f) was dominated by Mot, SubC and visual networks. The Mot and DMN exhibited the highest within-network connectivity, while the SubC-Mot, VI-Mot, and VII-Vas pairs formed the strongest between-network links. This prominence of Mot, SubC, and visual networks aligns with the Visual–Motor integration and attention switching demands of the TMT-B task.

### 3.4. Network Contribution and Connection Strengths

**Figure 5:**
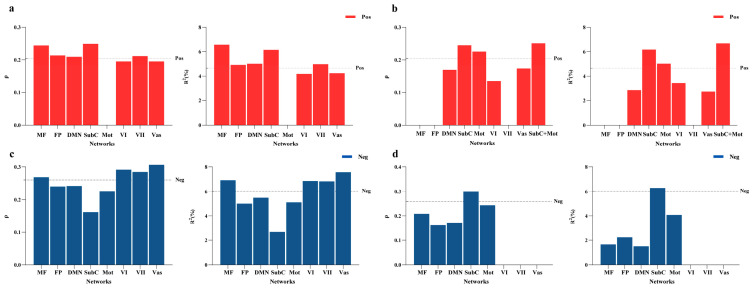
Virtual lesion and inclusion analyses. Network-specific contributions to predictive performance were evaluated using virtual lesion and inclusion analyses. In each panel, the left plot shows Spearman correlation (ρ) and the right plot shows the coefficient of determination (R^2^). The horizontal dotted line indicates the baseline performance of the full-brain CPM. (**a**,**b**) MoCA-pos model: (**a**) Virtual lesion analysis: performance after systematically removing one functional network at a time. (**b**) Inclusion Analysis: performance of models built using only specific isolated network. (**c**,**d**) TMT-B-neg model: (**c**) Virtual lesion analysis. (**d**) Inclusion analysis. Abbreviations: CPM, connectome-based predictive modeling; MF, Medial Frontal; FP, Frontoparietal; DMN, Default Mode Network; SubC, Subcortical Cerebellum; Mot, Motor; VI, Visual I; VII, Visual II; Vas, Visual Association.

**Figure 6:**
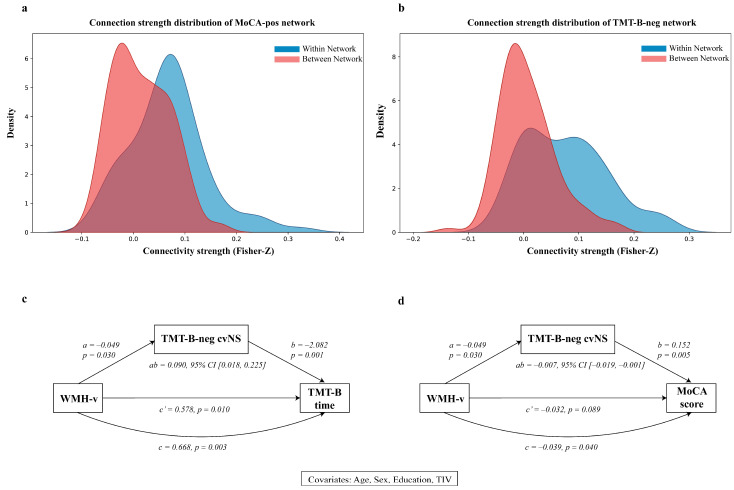
Connection strength distributions and mediation analysis. (**a**,**b**) Kernel density estimation of connection strengths for edges selected in the final predictive models. The *x*-axis represents connection strength after Fisher Z transformation, and the *y*-axis represents density. (**a**) MoCA-pos network. (**b**) TMT-B-neg network. (**c**,**d**) Mediation models. Models assessing whether TMT-B-neg cvNS statistically mediates the association between white matter hyperintensity volume (WMH-v) and cognitive outcomes. (**c**) TMT-B: TMT-B-neg cvNS partially mediated the association between WMH-v and TMT-B completion time; indirect effect *ab* = 0.090; 95% CI [0.018, 0.225]. (**d**) MoCA: TMT-B-neg cvNS showed a significant indirect effect on the association between WMH-v and MoCA score; indirect effect *ab* = −0.007; 95% CI [−0.019, −0.001]. All mediation models were adjusted for age, sex, education, and total intracranial volume. Abbreviations: CI, confidence interval; cvNS, cross-validated network strength; TMT-B, Trail-Making Test Part B; TMT-B-neg cvNS, cross-validated network strength of the TMT-B negative predictive network; TIV, total intracranial volume; WMH-v, white matter hyperintensity volume; MoCA, Montreal Cognitive Assessment.

To determine specific network contributions, we performed virtual lesion and inclusion analyses. For the MoCA-pos model, the Mot was found to be irreplaceable (Figure 5a), as its removal caused the CPM to fail in finding a significant predictive pattern (*p* > 0.05). Complementarily, a simplified model using only the SubC and Mot outperformed the full-brain baseline, suggesting the core predictive signal is highly concentrated within these networks (Figure 5b). Regarding topology, an analysis of connection strengths revealed that within-network connections exhibited significantly higher mean strengths (peaking approximately 0.1) compared to between-network connections (Figure 6a).

For the TMT-B-neg model, the SubC was the most critical component; its removal caused the largest performance drop (Figure 5c,d). This finding, combined with performance drops after lesioning the FP, DMN, and Mot, suggests that a distributed network including the Prefrontal–Subcortical–Cerebellar circuit may make an important contribution to executive function indexed by TMT-B, particularly visual attention and cognitive flexibility. Conversely, visual models failed to identify stable connections, suggesting that visual networks contribute primarily through interactions with other systems. Crucially, connection strength analysis revealed a distinct weak tie architecture (Figure 6b). Unlike the MoCA-pos model, the TMT-B-neg network was dominated by weak, between-network connections (peaking near zero), which accounted for 64.4% (105/163) of all predictive edges. This reliance on widespread, low-strength bridges aligns with the weak tie hypothesis, suggesting that executive control depends on integrating diverse brain regions through these weak connections [55].

### 3.5. Brain Behavior Correlation of NS

Partial Spearman correlation analyses controlling for age, sex, and education indicated that cvNS of the MoCA-pos network (MoCA-pos cvNS) was positively associated with MoCA scores (ρ = 0.175, *p* = 0.006), whereas cvNS of the TMT-B-neg network (TMT-B-neg cvNS) was negatively associated with TMT-B completion time (ρ = −0.227, *p* < 0.001). These results indicate that higher cvNS consistently corresponds to better cognitive performance.

However, in group comparisons, neither MoCA-pos cvNS nor TMT-B-neg cvNS showed significant differences between VRF-CN and VCI (VRF-CN vs. VCI; *p* = 0.166 and *p* = 0.223, respectively) or across the three clinical stages (VRF-CN, VaMCI, VaD; *p* = 0.244 and *p* = 0.110, respectively; Supplementary Figure S1). These findings suggest that cvNS may be more sensitive to interindividual variability in cognitive performance than to categorical clinical-stage differences, likely reflecting substantial within-stage heterogeneity.

In the external dataset, the NS demonstrated partial replication of the discovery findings. After adjusting for age, sex, and education, the final MoCA-pos NS was significantly associated with MoCA (ρ = 0.460, *p* = 0.006). The TMT-B-neg NS showed an attenuated and non-significant association with TMT-B time (ρ = −0.218, *p* = 0.215), with the direction consistent with the discovery dataset.

### 3.6. Mediation Analysis: TMT-B-Neg cvNS Shows Significant Indirect Effects Linking WMH with TMT-B and MoCA

The associations between WMH volume and cognition were first examined using Spearman correlations controlling for age, sex, education, and TIV. Overall, WMH was not significantly associated with MoCA scores (ρ = −0.102, *p* = 0.114), whereas higher WMH burden was associated with longer TMT-B completion time (ρ = 0.173, *p* = 0.007). In a subgroup analysis, WMH was significantly associated with MoCA in the VCI group (ρ = −0.242, *p* = 0.003), but not in the VRF-CN group (ρ = 0.131, *p* = 0.210).

We next examined whether WMH burden was associated with cvNS after controlling for age, sex, education, and TIV. WMH was not significantly associated with MoCA-pos cvNS (ρ = −0.074, *p* = 0.254), but was significantly associated with TMT-B-neg cvNS (ρ = −0.151, *p* = 0.018).

Mediation analyses with 5000 bootstrap iterations were then conducted to test whether cvNS mediated the associations between WMH burden and cognition, controlling for age, sex, education, and TIV. When MoCA-pos cvNS was entered as the mediator, no significant indirect effect was observed for either MoCA or TMT-B (MoCA: indirect = −0.002, 95% CI [−0.013, 0.006]; TMT-B: indirect = 0.010, 95% CI [−0.020, 0.114]).

In contrast, when TMT-B-neg cvNS was used as the mediator, significant indirect effects were observed for both outcomes. For TMT-B (Figure 6c), the indirect effect was significant (indirect = 0.090, 95% CI [0.018, 0.225], *p* = 0.023), while the direct effect remained significant (*p* = 0.010), consistent with a partial mediation pattern. The indirect pathway accounted for approximately 13.5% of the total association between WMH and TMT-B performance. For MoCA (Figure 6d), the indirect effect was also significant (indirect = −0.007, 95% CI [−0.019, −0.001], *p* = 0.031), whereas the direct effect was not significant (*p* = 0.089), consistent with an indirect-only mediation pattern. Together, these findings suggest that the TMT-B-neg connectivity pattern may capture a statistically significant indirect pathway linking WMH burden to cognitive performance.

### 3.7. Exploratory Classification Analysis

Exploratory classification analyses are reported in the Supplementary Materials and Results. Classification performance was modest and broadly comparable across models. Adding MoCA-pos cvNS had consistent but limited improvements; however, overall discrimination remained suboptimal. Given the modest discrimination, wide uncertainty, and exploratory nature of these analyses, the classification results should not be interpreted as evidence of a clinically deployable classifier or clinically applicable biomarker. The primary aim of this study was to identify cognition-related functional connectome patterns and examine their associations with cognitive performance, rather than to develop a diagnostic classifier.

## 4. Discussion

In this multicenter study, we applied CPM to identify FC signatures associated with cognitive performance across the VCI continuum. Our main findings include the following: First, CPM identified cross-validated connectivity patterns related to MoCA and TMT-B, predominantly centered on the Frontoparietal, Motor, and Subcortical Cerebellum regions. These patterns also showed topological specificity. In particular, the TMT-B-neg network was characterized by a high proportion of weak between-network connections. Second, cvNS was significantly associated with cognition in the discovery dataset. In the external dataset, the NS computed using the consensus masks from the discovery dataset showed an exploratory association with global cognition, as MoCA-pos NS was significantly associated with MoCA scores. Third, greater WMH burden was associated with worse TMT-B performance and cvNS, and mediation analyses suggested that cvNS computed from the TMT-B connectivity pattern identified by CPM captured a statistically significant indirect pathway linking WMH burden to both MoCA and TMT-B performance.

The MoCA-pos and TMT-B-neg networks derived from CPM showed consistent patterns across permutation and motion sensitivity analyses, supporting their distinct functional relevance. Specifically, the MoCA-pos network was characterized by extensive interconnections within and between the DMN, Mot, and SubC networks. In contrast, the TMT-B-neg network relied heavily on Motor–Subcortical–Frontoparietal coupling, reflecting the specific visuomotor switching, sequencing, and attentional control demands of the TMT-B task [56,57]. Furthermore, our network topography and virtual lesion analyses may offer functional support for the hypothesis of structural hub failure in VCI [58,59,60]. These findings align with prior work linking executive deficits in VCI to the disruption of Prefrontal–Subcortical–Cerebellar circuits, underscoring that distinct yet overlapping functional architectures are recruited to support different cognitive domains [61,62,63].

Although the MoCA-pos and TMT-B-neg models showed model-specific significance, their cross-validated explained variance was modest (R^2^ = 4.64% and 6.03%, respectively). These findings indicate that the identified functional connectivity patterns capture only a limited component of cognitive heterogeneity across the VCI continuum. The remaining variance may reflect unmeasured factors, such as white matter microstructural damage, neurodegenerative pathology, cognitive reserve, and measurement variability. Accordingly, these models should be interpreted as exploratory network-level correlates rather than clinically applicable predictive biomarkers.

Crucially, we used participant-wise cvNS as an interpretable continuous measure for all subsequent inferential analyses within the discovery dataset. In contrast to NS computed using a mask derived from the full discovery dataset, cvNS provides an out-of-sample estimate and therefore represents a more conservative approach, which would be expected to show smaller effect sizes. Nevertheless, higher cvNS was associated with better cognitive performance. By contrast, group comparisons of cvNS across clinical stages were not significant. This pattern is not necessarily inconsistent, given the heterogeneity of VCI, including variation in VRFs and pathological burdens, as well as the relatively limited size of the VaD subgroup. Collectively, these findings suggest that cvNS is more appropriately conceptualized as a continuous functional measure associated with interindividual cognitive variability, rather than as a measure for distinguishing categorical clinical stages in the current sample.

In the external dataset, we assessed the CPM-derived connectivity patterns in an exploratory external setting by applying fixed consensus masks derived from the discovery dataset to compute NS in each external participant using the same pipeline. We observed a significant association between MoCA-pos NS and MoCA scores, providing exploratory external support for the MoCA-related connectivity pattern despite differences in clinical context, sample characteristics, and image acquisition. In contrast, the association between TMT-B-neg NS and TMT-B did not reach statistical significance, which is not unexpected. TMT-B completion time is influenced by visuomotor speed, upper limb function, and stroke-related deficits. In this external dataset of patients with acute mild ischemic stroke, these noncognitive factors may disproportionately prolong completion time, thereby increasing measurement noise and reducing sensitivity to the relevant network. Accordingly, the external findings should be interpreted as preliminary and exploratory, rather than as evidence of formal external validation.

Furthermore, our edge distribution analysis showed that a substantial portion of the predictive information, particularly for TMT-B performance, was carried by weak between-network connections (64.4%). This topological feature is consistent with the weak tie hypothesis in complex network theory, which proposes that low strength links connecting different networks may serve as important bridges that support communication and integration across distributed brain regions [55,64,65]. In the context of VCI, this pattern suggests that executive dysfunction may reflect a breakdown of large-scale functional integration, rather than disruption of only a few strong pathways.

In our study, higher WMH burden was significantly associated with worse TMT-B performance. However, unlike some prior reports, we did not observe a significant overall association between WMH volume and MoCA. We attribute this discrepancy to the age characteristics of our dataset, which is relatively younger (mean age 62 years) compared to typical dementia studies (often >65 years) [66,67]. Given that WMH burden is strongly age-dependent [68], the direct correlation between lesion volume and global cognitive decline may be attenuated in relatively younger or earlier stage populations compared to older cohorts. This pattern aligns with other studies in younger populations, suggesting that the cognitive effects of WMH may not yet be fully manifest or may be partially buffered by cognitive reserve [69,70]. Our subgroup analysis provides additional support for this interpretation, WMH burden was significantly associated with MoCA in the VCI group, but not in the VRF-CN group.

Although WMH is a well-established predictor of cognitive decline, our findings further suggest that part of this association may operate through the disruption of functional network association [71,72]. Specifically, TMT-B-neg cvNS statistically mediated the association between WMH burden and both TMT-B completion time and MoCA score. For TMT-B, the mediation was partial, accounting for approximately 13.5% of the total association. For MoCA, we observed an indirect-only pattern, suggesting that WMH may be linked to global cognition specifically through the disruption of the specific TMT-B-neg functional connectivity pattern [70,73,74]. This aligns with the clinical characterization of VCI as a fundamentally dysexecutive syndrome, where global deficits are often secondary to executive failure [29,75]. More broadly, these findings are consistent with the disconnection hypothesis, whereby WMH-related lesions may compromise the long-range tracts that support distributed functional coordination, contributing to network-level disintegration and subsequent executive dysfunction [14]. At the same time, the persistence of a significant direct effect for TMT-B indicates that WMH may also relate to cognition through additional processes not captured by this specific functional network, such as global atrophy, cortical thinning, or the disruption of other neural systems. Together, these results suggest that vascular pathology likely influences cognition through multiple parallel pathways rather than a single mechanism.

This study has several limitations. First, the cross-sectional design limits our ability to make strict causal inferences regarding disease progression. Although the mediation analyses were consistent with an indirect statistical association among structural pathology, functional network disruption, and cognitive impairment, they cannot establish biological causality or exclude bidirectional relationships. Longitudinal studies are therefore needed to clarify the temporal sequence of these associations. Second, despite rigorous motion control procedures, fMRI data in elderly populations are inherently susceptible to noise. Third, the absence of significant group-wise differences in cvNS may reflect limited statistical power in smaller subgroups, particularly the VaD group, as well as substantial heterogeneity within clinical stages. Fourth, the external dataset was limited in size and was not strictly situated within the VCI continuum, as the acute phase may have affected the accuracy of cognitive assessment. Therefore, the external findings should be interpreted cautiously, and future studies should evaluate the consensus masks and the corresponding network strength measures in larger and clinically better matched external cohorts. Finally, future multimodal studies incorporating diffusion MRI are needed to provide complementary structural validation. Tract-specific diffusion measures and structural connectomes could be used to determine whether weak between-network functional edges correspond to compromised long-range white matter pathways. Quantifying structure–function coupling may further clarify whether these functional patterns reflect structural disconnection, compensatory reorganization, or both.

## 5. Conclusions

In conclusion, this study provides new evidence linking CPM-identified functional connectivity patterns to cognitive performance across the VCI continuum. Mediation analyses further indicated an indirect statistical association among WMH burden, the TMT-B-related connectivity pattern, and both global cognition and TMT-B performance. In addition, the TMT-B-neg network showed a distinct topological organization, with a substantial contribution from weak between-network connections, supporting a potential role of large-scale functional integration for cognitive flexibility in VCI. Together, these findings suggest that distributed functional disconnection may represent a network-level correlate of cognitive impairment across the VCI continuum.

## Figures and Tables

**Table 1:** Demographic, clinical, and neuropsychological characteristics of the study dataset.

Characteristic	Number (%) or Mean (Standard Deviation, Range)		
	Total (*N* = 247)	VRF-CN (*N* = 97)	VCI (*N* = 150)
Age, years	62.22 (6.32, 50–76)	60.88 (6.41, 51.00–74.00)	63.09 (6.13, 50–76)
Education, years	12.84 (3.16, 6–23)	14.22 (2.82, 8–23)	11.95 (3.06, 6–23)
Sex, female, ***n*** (%)	114 (46.15%)	48 (49.48%)	66 (44.00%)
BMI, kg/m^2^	24.52 (2.90, 18.75–34.41)	24.67 (3.25, 19.15–34.41)	24.42 (2.65, 18.75–30.86)
History of smoking, ***n*** (%)	107 (43.32%)	35 (36.08%)	72 (48.00%)
Physical exercise, ***n*** (%)	186 (75.30%)	72 (75.00%)	114 (76.00%)
Coronary heart disease, ***n*** (%)	22 (8.91%)	4 (4.12%)	18 (12.00%)
History of hypertension, ***n*** (%)	182 (73.68%)	65 (67.01%)	117 (78.00%)
History of diabetes, ***n*** (%)	106 (42.91%)	39 (40.21%)	67 (44.67%)
Hyperlipidemia, ***n*** (%)	159 (64.37%)	73 (75.16%)	86 (57.33%)
Systolic blood pressure, mmHg	133.09 (15.93, 75–170)	131.24 (16.31, 100–170)	134.29 (15.61, 75–169)
Diastolic blood pressure, mmHg	81.11 (11.38, 53–111)	81.27 (12.08, 59–110.50)	81.00 (10.94, 53–110)
Fasting blood glucose (mmol/L)	6.26 (3.66, 2.81–52)	6.28 (4.87, 3.80–52)	6.25 (2.62, 2.81–28.53)
Total cholesterol (mmol/L)	4.54 (0.96, 2.13–7.69)	4.63 (0.91, 2.75–6.96)	4.48 (1.00, 2.13–7.69)
Triglycerides (mmol/L)	1.77 (1.18, 0.38–11.69)	1.79 (1.09, 0.53–7.27)	1.75 (1.24, 0.38–11.69)
High-density lipoprotein (mmol/L)	1.25 (0.36, 0.61–3.16)	1.29 (0.38, 0.67–3.16)	1.22 (0.34, 0.61–3.16)
Low-density lipoprotein (mmol/L)	2.65 (0.76, 0.99–5.10)	2.71 (0.71, 1.42–4.85)	2.62 (0.79, 0.99–5.10)
MoCA	23.17 (3.54, 14–30)	26.56 (1.51, 20–30)	20.98 (2.64, 14–24)
MMSE	27.60 (2.01, 20–30)	28.61 (1.25, 24–30)	26.94 (2.14, 20–30)
TMT-A time	53.06 (21.67, 18.66–130.47)	46.28 (18.37, 18.66–129.50)	57.45 (22.55, 23.50–130.47)
TMT-B time	83.72 (39.37, 29.69–300)	70.17 (25.44, 29.69–160)	92.48 (44.10, 37.22–300)
CAVLT	38.78 (9.16, 15–68)	42.12 (9.23, 25–68)	36.61 (8.46, 15–56)
White matter hyperintensity volume (mL)	7.50 (11.46, 0.00–80.14)	6.21 (9.56, 0–72.82)	8.33 (12.50, 0.02–80.14)

Data are presented as mean (standard deviation, range) or *n* (%). Abbreviations: BMI, Body Mass Index; MoCA, Montreal Cognitive Assessment; MMSE, Mini-Mental State Examination; TMT-A, Trail-Making Test A; TMT-B, Trail-Making Test B; CAVLT, Chinese Auditory Verbal Learning Test.

## Data Availability

The data are not publicly available due to privacy and ethical restrictions involving human participants. Anonymized data will be available on reasonable request considered by the corresponding author.

## Supplementary Materials

The following supporting information can be downloaded at: https://www.mdpi.com/article/10.3390/brainsci16070695/s1, Supplementary Methods [76,77,78]; Supplementary Results; Figure S1: Group comparisons of cross-validated network strength; Table S1: Demographic, Clinical, and Neuropsychological Characteristics of the external dataset; Table S2: Results of normality tests for behavioral variables; Table S3: CPM results; Table S4: Correlations between FD-Jenkinson and behavioral variables (neuropsychological assessments); Table S5: Edge overlap between original and motion corrected CPMs after including FD-Jenkinson as a covariate; Table S6: Exploratory classification performance.

## Abbreviations

The following abbreviations are used in this manuscript:

CPM	Connectome-based predictive modeling
LOOCV	Leave-one-out cross-validation
cvNS	Cross-validated network strength
TMT-B-neg cvNS	Cross-validated network strength of the TMT-B negative predictive network
TIV	Total intracranial volume
WMH-v	White matter hyperintensity volume
VCI	Vascular cognitive impairment
BOLD	Blood Oxygen Level-Dependent
FD	Framewise displacement
MoCA	Montreal Cognitive Assessment
TMT-B	Trail-Making Test Part B
MNI152	Montreal Neurological Institute 152 template
MF	Medial Frontal
FP	Frontoparietal
DMN	Default Mode Network
SubC	Subcortical Cerebellum
Mot	Motor
VI	Visual I
VII	Visual II
Vas	Visual Association
CI	Confidence interval
