# Supplementary material for: Functional Connectome Predicts Cognition and Links White Matter Hyperintensity Burden to Cognitive Impairment Across the Vascular Cognitive Impairment Continuum
Source: Brain Sci. 2026 Jun 30;16(7):695. doi: 10.3390/brainsci16070695 (PMC13406254; doi:10.3390/brainsci16070695)
Supplement: Supplementary file 1 [file brainsci-16-00695-s001.zip › brainsci-4370002-supplementary.pdf]

brain sciences

1. Supplementary Methods

1.1. MRI data acquisition

For the discovery dataset, the resting-state fMRI (rs-fMRI) parameters for the SIEMENS Trio Tim were: Repetition Time (TR) = 2000 ms, Echo Time (TE) = 30 ms, and Slice Thickness = 3.8 mm. The fMRI scanning protocol for the GE 750W was upgraded during the acquisition period. The initial protocol used TR = 2000 ms, TE = 25 ms, and Slice Thickness = 5 mm, while the upgraded protocol used TR = 2000 ms, TE = 30 ms, and Slice Thickness = 3.8 mm, matching the SIEMENS parameters. For structural T1-weighted images, the SIEMENS Trio Tim used a 3D MPRAGE sequence with the following parameters: TR = 6.7 ms, TE = 2.26 ms, flip angle = 9°, and voxel size = 1×1×1 mm^3. The GE 750W used a 3D BRAVO sequence with these parameters: TR = 8.2 ms, TE = 3.1 ms, flip angle = 12°, and voxel size = 0.94×0.94×0.5 mm^3. For the external dataset, multimodal MRIs were acquired on a 3T GE SIGNA Architect scanner. The rs-fMRI parameters were as follows: TR = 3150 ms, TE = 30 ms, and Slice Thickness = 3 mm while the parameters for the T1-weighted images were: TR = 7.7 ms, TE = 3.1 ms, flip angle = 12°, and voxel size = 0.5×0.5×1 mm^3.

1.2. Exploratory classification analysis

As an exploratory analysis, we evaluated whether cross-validated NS (cvNS) provides incremental discriminative value for classifying participants as VRF-CN versus VCI. Four algorithms were used, including Logistic Regression (LR), Random Forest (RF)[76], Support Vector Machine (SVM)[77], and eXtreme Gradient Boosting (XGBoost)[78]. The MoCA-pos cvNS, TMT-B-neg cvNS, WMH volume, age, sex, and years of education were used as the input features for the machine learning models. We used a nested repeated stratified cross-validation framework (outer 5-fold × 10 repeats; 50 outer evaluations). To strictly prevent data leakage, all preprocessing steps including feature scaling and hyperparameter tuning (Randomized Search for XGBoost; Grid Search for others) were confined within the inner loops. Model performance was evaluated using ROC-AUC, PR-AUC, and weighted F1-score, with 95% confidence intervals derived from the empirical 2.5th–97.5th percentiles of the 50 outer-loop iterations. All machine learning analyses were performed using the Scikit-learn package (v1.2.1).

2. Supplementary Results

2.1. Exploratory classification analysis

Overall discriminative performance was modest and broadly comparable across algorithms and feature sets. The best-performing configuration was a Random Forest model using demographics (age, sex, and years of education) plus MoCA-pos cvNS, achieving a mean ROC-AUC of 0.727 with an empirical 95% CI of 0.575–0.862, PR-AUC of 0.812 (0.678–0.914), and weighted F1-score of 0.684 (0.573–0.776). Adding WMH and/or TMT-B-neg cvNS did not yield material improvements over this baseline (Supplementary Table 6). Given the modest specificity-sensitivity trade-offs and the exploratory nature of this analysis, these results should be interpreted as preliminary support for incremental information content rather than a clinically deployable classifier.

Boxplots show the distribution of cvNS for the MoCA-pos and TMT-B-neg networks across clinical groups. No significant group differences were observed in the binary comparison between the VRF-CN and VCI groups (a–b), or across the three clinical stages of VRF-CN, VaMCI, and VaD (c–d). **Abbreviations**: cvNS, cross-validated network strength; MoCA, Montreal Cognitive Assessment; TMT-B, Trail Making Test Part B; VRF-CN,

vascular risk factors with normal cognition; VCI, vascular cognitive impairment; VaMCI, vascular mild cognitive impairment; VaD, vascular dementia.

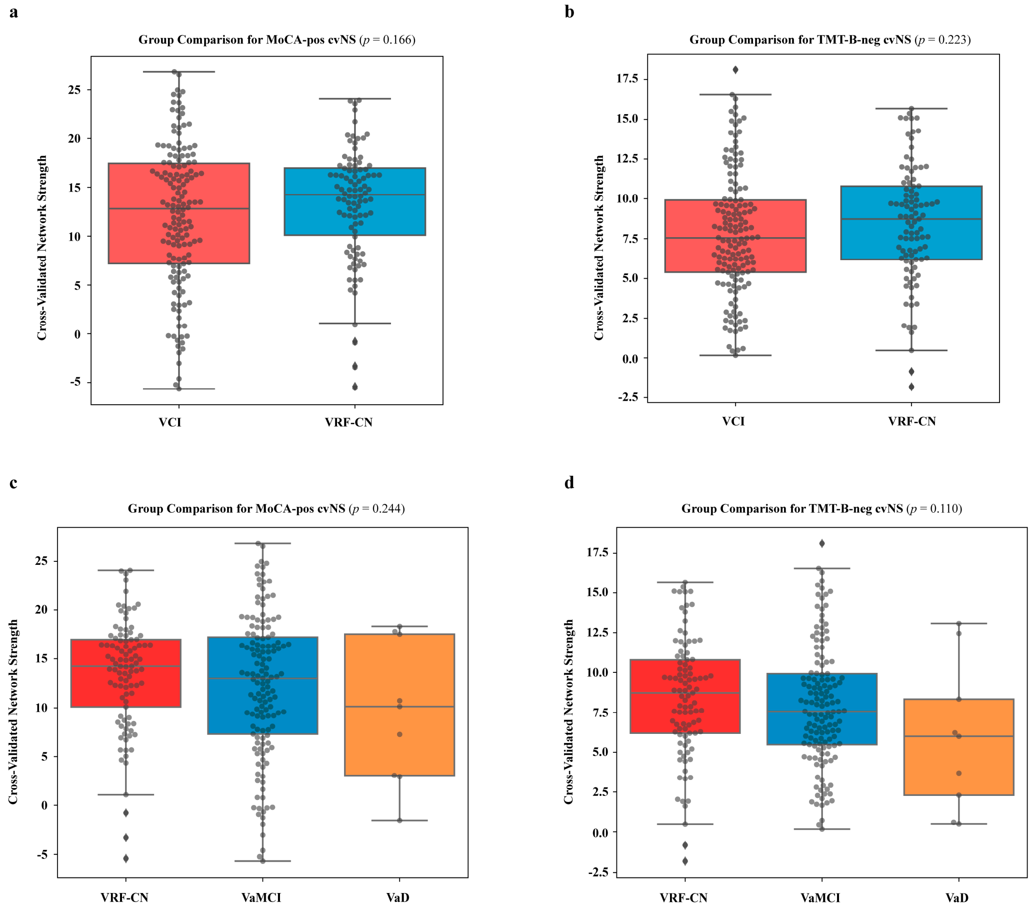

Supplementary Figure S1. **Group comparisons of cross-validated network strength.**

Supplementary Table S1. Demographic, Clinical, and Neuropsychological Characteristics of the external dataset

Characteristic	Number (%) or Mean (Standard Deviation, Range)（N = 37）
**Age, years**	59.75（9.16, 40.00-74.00）
**Education, years**	9.03 (2.79, 5.00-15.00)
**Sex, Female, n (%)**	11 (29.7%)
**BMI, kg/m2**	24.79 (3.06, 17.90-30.20)
**History of smoking, n (%)**	19 (51.4%)
**Coronary heart disease, n (%)**	4 (10.8%)
**History of hypertension, n (%)**	31 (83.8%)
**History of diabetes, n (%)**	7 (18.9%)
**Hyperlipidemia, n (%)**	21 (56.8%)
**Systolic blood pressure, mmHg**	148.50 (12.81, 111.00-173.00)
**Diastolic blood pressure, mmHg**	86.01 (8.82, 70.00-109.00)
**MoCA**	19.22 (5.86, 4.00-28.00)
**TMT-A**	89.76 (39.79, 38.00-150.00)

TMT-B	167.14 (92.42, 55.00-300.00)

Supplementary Table S2: Results of normality tests for behavioral variables

Neuropsychological assessments	K-S statistic	p - value
**MoCA**	0.107	< 0.001
**MMSE**	0.175	< 0.001
**TMT-A**	0.109	< 0.001
**TMT-B**	0.182	< 0.001
**CAVLT**	0.069	0.006

Supplementary Table S3: CPM results

CPM	ρ	R^2 (%)	*p-perm*	MAE	ComConn
**MoCA - pos**	0.202	4.64	0.034	2.90	223
**MoCA - neg**	0.193	3.48	0.048	2.97	205
**MMSE - pos**	0.068	0.06	0.284	1.63	105
**MMSE - neg**	-0.016	0.55	0.547	1.66	130
**TMT-B - pos**	0.049	0.39	0.340	30.51	160
**TMT-B - neg**	0.260	6.03	0.001	28.39	163
**TMT-A - pos**	0.059	0.24	0.322	17.52	184
**TMT-A - neg**	0.056	0.26	0.325	17.41	145
**CAVLT - pos**	-0.276	7.33	0.969	8.75	49
**CAVLT - neg**	-0.049	0.21	0.646	8.26	60

p_perm denotes the model-specific permutation p value. The p values were not adjusted across cognitive outcomes or CPM models because each model was interpreted separately rather than as a surrogate test of a single omnibus null hypothesis. Abbreviations: ComConn, Common Connections; p-perm, p-value derived from a 5,000-permutation test.

Supplementary Table S4: Correlations between FD-Jenkinson and behavioral variables (neuropsychological assessments)

Neuropsychological assessments	MoCA	MMSE	TMT-A	TMT-B	CAVLT
**r**	0.076	0.055	-0.047	0.031	0.058
***p* - value**	0.235	0.387	0.460	0.620	0.367

Supplementary Table S5: Edge overlap between original and motion corrected CPMs after including FD-Jenkinson as a covariate

Abbreviations: ComConn, Common Connections; OrigConn, Original Connections

CPM	MoCA - pos	MoCA - neg	TMT-B - pos	TMT-B - neg
**Sharing ComConn**	217	189	148	145
**OrigConn**	223	205	160	163
**Overlap**	97.31%	92.20%	92.50%	88.96%

Supplementary Table S6: Exploratory classification performance (nested repeated CV): ROC-AUC (mean [95% Confidence Interval])

RF	SVM	LR	XGBoost

**A**	0.706 [0.580–0.819]	0.687 [0.547–0.804]	0.711 [0.568–0.814]	0.698 [0.570–0.819]
**B**	0.696 [0.582–0.822]	0.690 [0.566–0.800]	0.707 [0.581–0.810]	0.671 [0.556–0.803]
**C**	0.727 [0.575–0.862]	0.688 [0.570–0.794]	0.707 [0.586–0.809]	0.702 [0.571–0.816]
**D**	0.690 [0.560–0.794]	0.686 [0.546–0.808]	0.704 [0.562–0.810]	0.669 [0.547–0.776]
**E**	0.703 [0.563–0.822]	0.694 [0.550–0.799]	0.702 [0.582–0.825]	0.680 [0.551–0.780]
**F**	0.713 [0.562–0.839]	0.679 [0.564–0.801]	0.702 [0.591–0.826]	0.680 [0.536–0.815]
**G**	0.675 [0.549–0.800]	0.684 [0.545–0.796]	0.702 [0.555–0.825]	0.654 [0.514–0.763]
**H**	0.695 [0.553–0.829]	0.690 [0.557–0.775]	0.699 [0.584–0.825]	0.674 [0.539–0.815]

CIs are empirical percentiles across 50 outer-loop evaluations (5-fold × 10 repeats); Abbreviations: RF, Random Forest; SVM, Support Vector Machine; LR, Logistic Regression; XGBoost, eXtreme Gradient Boosting.

Feature set: A: Demographic variables (age, sex, and years of education);
B: Demo + WMH;
C: Demo + MoCA-pos cvNS;
D: Demo + TMT-B-neg cvNS;
E: Demo + MoCA-pos cvNS + TMT-B-neg cvNS
F: Demo + WMH + MoCA-pos cvNS;
G: Demo + WMH + TMT-B-neg cvNS;
H: Demo + WMH + MoCA-pos cvNS + TMT-B-neg cvNS.

https://doi.org/10.3390/brainsci16070695
